# A guarded light pipe for direct visualization during primary scleral buckling on the Ngenuity platform

**DOI:** 10.1186/s40942-020-00246-9

**Published:** 2020-09-18

**Authors:** Joshua S. Agranat, Vivian P. Douglas, Konstantinos A. A. Douglas, John B. Miller

**Affiliations:** Retina Service, Massachusetts Eye and Ear, Department of Ophthalmology, Harvard Medical School, Boston, MA USA

**Keywords:** Scleral buckle, Intraoperative visualization, Three-dimensional digital display, Retina

## Abstract

**Background:**

Visualization during scleral buckling is traditionally achieved via indirect ophthalmoscopy. Recent advances have utilized the surgical microscope and a 25 gauge cannula-based endoillumination system, also known as a Chandelier lighting system. This report details an improved approach using a guarded 25 or 27 gauge light pipe and the Ngenuity digital three dimensional platform.

**Methods:**

A standard Alcon light pipe is modified with a silicone guard to expose only 5 mm of the tip of the light pipe. The guard is created from the silicone that is already opened to secure the ends of the encircling band most often employed sleeve (e.g. 70, 270). This guarded light pipe is then inserted into the cannula as an alternative to a Chandelier lighting system.

**Results:**

This is a technical report of a surgical visualization technique using a three dimensional digital visualization platform with a modified handheld vitrectomy light pipe.

**Conclusion:**

The utilization of a guarded light pipe for visualization during primary scleral buckling is a promising, effective, and efficient technique. The three dimensional digital display allows for better educational impact and surgical communication with trainees and ancillary members of the surgical team.

## Background

Scleral buckling is a method for retinal detachment repair that has been employed for nearly 60 years, first pioneered Schepens [1]. Despite the widespread adoption of Dr. Robert Machemer’s pars plana vitrectomy [2], scleral buckling still plays an important role in the repair of retinal detachment in properly selected patients. Primary scleral buckling without concurrent vitrectomy remains most helpful in younger patients without a posterior vitreous detachment, with formed vitreous, extensive peripheral lattice degeneration, inferior pathology, and/or chronic subretinal fluid.

The primary scleral buckling procedure involves localization of all the breaks and scleral marking the site of the breaks. Next typically the retinal pigment epithelium and choroid are irritated via cryotherapy under indirect ophthalmoscopy to induce chorioretinal adhesions. A segmental or encircling element is sutured to the sclera and the height of the element is monitored and adjusted. Depending on the characteristics of the detachment trans-scleral subretinal fluid drainage may be performed.

Traditionally, visualization during scleral buckling relies completely on an indirect ophthalmoscope to localize and apply cryotherapy to the breaks, confirm adequate subretinal fluid drainage, and adjust the buckle height. Recent advances in visualization have utilized the surgical microscope and a 25 gauge cannula-based endoillumination system, also known as a Chandelier lighting system [3]. The Chandelier is attached using a semi-flexible cable with limited ability to directionally illuminate the peripheral retina 360 degrees. While it can be manipulated somewhat, the ergonomics and security of the surgeon’s hand position are inferior to the vitrectomy light pipe we use during every vitrectomy case.

Herein, we report a modified scleral buckling visualization approach using a guarded 25 or 27 gauge light pipe and the Ngenuity digital three dimensional platform. The trimmed end of the Watzke-Allen sleeve slides up the shaft of the light pipe as guard to prevent insertion into the vitreous past just the internal os of the cannula, trying to minimize vitreous dragging and thus iatrogenic breaks.

## Surgical Technique

The procedure was performed using the Ngenuity digital three-dimensional (3D) visualization system [4] which relays overlapping real-time video obtained from the surgical microscope into a display monitor at the foot of the patient’s bed; stereopsis is appreciated when the surgeon, assistants and nursing team wears polarizing glasses. A peritomy was performed to expose the sclera. The rectus muscles were isolated with 4–0 silk sutures. A single 27-gauge trocar canula was inserted 3.5 to 4 mm from the limbus through the pars plana. The trocar is most often placed superiorly in the hemisphere opposite the greatest amount of pathology and planned location of external drainage to ease bimanual manipulation by the surgeon at 12 o’clock (e.g. superonasal trocar for illumination if the drainage site is planned for and the retinal holes are most present inferotemporally).

**Fig. 1:**
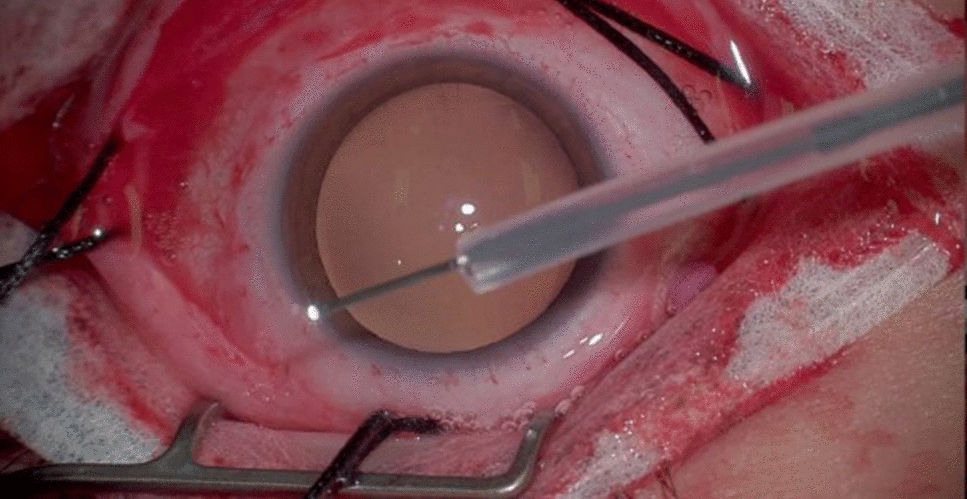
Standard vitrectomy light pipe with silicone guard leaving only 5 mm of the tip exposed (see Additional file 1)

A standard Alcon light pipe is modified with a silicone guard to expose only 5 mm of the tip of the light pipe (Fig. 1). The guard is created from the silicone sleeve (e.g. 70, 270) that is already opened to secure the ends of the encircling band most often employed and cut to allow only 5 mm of the light pipe to be exposed. This guarded light pipe is then inserted into the cannula and a careful 360-degree scleral depressed examination is performed using the 3D visualization system to identify all breaks and the extent of the retinal detachment. After identification, all the breaks are treated with focal cryotherapy and the amount of freezing was directly monitored and titrated by both supervising physician and trainee. The scleral band or buckle is then secured in all intended quadrants without the microscope (as traditionally done). Direct visualization with the guarded light pipe can be used to either watch active subretinal drainage with a subretinal needle or to ascertain the amount of subretinal fluid if an external approach is done without the microscope. The buckle was secured in the desired position at the equator and the height was modified. An anterior chamber paracentesis decreased the intraocular pressure in preparation for the scleral buckle. The sclerotomy was sutured after removal of the cannula and the conjunctiva was reapproximated.

## Discussion

The core aspects of the scleral buckle procedure have gone unmodified since Lincoff reported the use of cryotherapy with scleral silicon sponge placement in 1965 [5]. Since this report, visualization of the breaks and the position and height of the buckle has been predominately achieved with indirect ophthalmoscopy. In 2013, Nam et al. reported the use of a chandelier endoilluminator coupled to the surgical microscope [3]. This technique allowed the surgeon to directly visualize the fundus and forgo the inconvenience and time needed to repeatedly adorn the indirect ophthalmoscope. It also allowed both surgeon and assistant to more closely approximate the same view of the intraoperative steps. The use of a trocar canula was an elegant approach which provided easy access for the chandelier light with minimal trauma to the sclera or vitreous. The efficacy and safety of this procedure has been reproduced in reports of successful scleral buckling using chandelier illumination [6–9]. Decreased case duration, improved primary reattachment rate and visual acuity, and augmented educational value of the chandelier assisted scleral buckle was reported by Narayanan et al. in 2016 [10]. Kimura and colleagues engineered a flexible chandelier fiber probe holder which provided some ability to control and direct the illuminating probe [11]. Other chandelier probes exist on the market in 25 and 27 gauge instrumentation including those from Synergetics, DORC, and Alcon Laboratories Inc. In general, the ergonomics and stability of the chandelier remain inferior to the light pipe routinely used for vitrectomy. The use of a standard light pipe may also result in cost savings compared to chandelier systems.

This report offers another iterative improvement in the progression of scleral buckling in the use of a guarded light pipe and a 3D visualization system. The guard on the light pipe is made of silicone and only exposes the distal 5 mm of the light pipe tip, just slightly more than the length of the cannula. We have used both a 25 and 27 gauge canula based on availability at the time of repair. The guard stops the probe from inadvertently dragging vitreous and likely increases the safety margin of the procedure. It also offers the vitreoretinal surgeon a familiar instrument while maximizing control of light location and directionality. Manipulation of the light pipe permits directed illumination for 360 degree examination and cryotherapy via a single canula.

The 3D visualization system has many benefits over the traditional optical microscope. The most significant benefits particular to this procedure are the use of lower illumination levels [12], improved depth of field [13], excellent trainee and staff viewing [14], ergonomics [15], high recording quality, and more efficient and shorter cases compared to using an indirect ophthalmoscope [10].

In the academic setting the ability for the attending and trainee to simultaneously visualize the cryotherapy, external drainage of subretinal fluid, and buckle height adjustment is a tremendous learning experience. To date, the technique reported herein has been trialed with six different vitreoretinal fellows participating in the surgery. Of additional benefit, several residents pursuing vitreoretinal fellowship also directly observed the key steps of the primary scleral buckle in 3D.

The safety of this modification has not been formally evaluated. There has been one prior report of endophthalmitis with the chandelier endoillumination system [16]. While we only have early experience with our described technique, we have had no adverse events or outcomes associated with this procedure. Furthermore, we would not expect that the rates of infection or other complications would be higher than when using chandelier endoillumination. More cases are needed in order to confirm safety of the technique.

In summary, the guarded light pipe for primary scleral buckling is a promising, effective, and efficient technique. The combination of the 3D digital display augments the educational impact and enhances surgical communication.

## Supplementary information

**Additional file 1.** Standard vitrectomy light pipe with silicone guard leaving only 5 mm of the tip exposed.

## Data Availability

All data generated or analyzed during this study are included in this published article.
